# Effect of aspirin on maternal hemodynamics in Chinese women at high risk for preterm pre‐eclampsia: longitudinal study

**DOI:** 10.1002/uog.70027

**Published:** 2025-09-12

**Authors:** X. Wang, J. Lin, Y. Chen, I. S. Wong, J. Liu, F. Liu, S. L. Lau, Q. Zhang, X. Xu, D. S. Sahota, A. P. W. Lee, L. C. Poon

**Affiliations:** ^1^ Department of Obstetrics and Gynecology, Nanchong Central Hospital The Second Clinical Medical College of North Sichuan Medical University Sichuan China; ^2^ Department of Obstetrics and Gynecology The Chinese University of Hong Kong Hong Kong SAR China; ^3^ Department of Obstetrics and Gynecology Fujian Maternity and Child Health Hospital Fujian China; ^4^ Shenzhen Research Institute The Chinese University of Hong Kong Hong Kong SAR China; ^5^ Department of Medicine & Therapeutics, Prince of Wales Hospital The Chinese University of Hong Kong Hong Kong SAR China

**Keywords:** aspirin, cardiac output, heart rate, hemodynamic, high‐risk, mean arterial pressure, pre‐eclampsia, stroke volume, systemic vascular resistance

## Abstract

**Objectives:**

To compare the maternal hemodynamics of Chinese women at low and at high risk for preterm pre‐eclampsia (PE) and assess the differences in hemodynamic parameters between high‐risk women with or without prophylactic aspirin.

**Methods:**

This was a prospective longitudinal case–control study of 857 Chinese women with a singleton pregnancy who participated in the first‐trimester screen‐and‐prevent program for PE at the Prince of Wales Hospital, Hong Kong SAR, China, between February 2020 and March 2023. The risk of developing preterm PE (delivery before 37 weeks) was determined using the Fetal Medicine Foundation combined test (maternal factors combined with mean arterial pressure (MAP), uterine artery pulsatility index and placental growth factor). The study population comprised three groups of women: (1) women at high risk (adjusted risk ≥ 1:100) for preterm PE who received 100 mg or 160 mg of aspirin according to maternal weight (< 40 kg or ≥ 40 kg, respectively), starting before 16 weeks' gestation until 36 weeks' gestation, until delivery or until PE was diagnosed if before 36 weeks; (2) women at high risk for preterm PE who did not receive aspirin; and (3) women at low risk (adjusted risk < 1:100) for preterm PE who were matched 1:1 to high‐risk women, according to maternal age, weight and date of the scan. MAP was measured using a device validated for use in pregnancy, and heart rate (HR), stroke volume (SV), cardiac output (CO) and systemic vascular resistance (SVR) were evaluated using two‐dimensional transthoracic echocardiography at 12 + 0 to 15 + 6 weeks, 20 + 0 to 24 + 6 weeks and 30 + 0 to 37 + 6 weeks' gestation. Log_10_ transformation was applied to fit the data to a Gaussian distribution. An adjusted multilevel linear mixed‐effects analysis was performed to compare the longitudinal changes of maternal hemodynamics across gestation between the three study groups.

**Results:**

This study comprised 389 women at low risk of preterm PE, 379 women at high risk of preterm PE who received aspirin and 89 women at high risk who did not receive aspirin. There was no significant difference in the estimated marginal mean (EMmean) of log_10_ HR across gestation among the three study groups. Compared with the low‐risk group, both high‐risk groups (with and without aspirin) exhibited consistently higher EMmean of log_10_ MAP and log_10_ SVR, and lower EMmean of log_10_ CO and log_10_ SV throughout gestation (all *P* < 0.001). Although maternal hemodynamic trajectories differed across the EMmean of log_10_ SV, CO, MAP and SVR between high‐risk women with aspirin and those without, there were no significant differences in these parameters between the two high‐risk groups.

**Conclusion:**

This study highlights the significant differences in maternal hemodynamic adaptation during pregnancy between Chinese women at high risk and those at low risk for preterm PE. Compared with low‐risk women, high‐risk women exhibited increased MAP and SVR, along with reduced SV and CO as early as the first trimester, and these alterations persisted throughout gestation. Notably, aspirin prophylaxis showed a limited effect on improving maternal hemodynamics in women at high risk for preterm PE, highlighting the need for alternative strategies to address the hemodynamic maladaptation in high‐risk women. © 2025 The Author(s). *Ultrasound in Obstetrics & Gynecology* published by John Wiley & Sons Ltd on behalf of International Society of Ultrasound in Obstetrics and Gynecology.

## INTRODUCTION

Women who are predisposed to develop preterm pre‐eclampsia (PE) are characterized by maternal cardiovascular maladaptation during pregnancy, as evidenced by decreased stroke volume (SV) and cardiac output (CO), along with increased systemic vascular resistance (SVR) and mean arterial pressure (MAP)^1^, ^2^, ^3^, ^4^, ^5^. The ASPRE (Combined Multimarker Screening and Randomized Patient Treatment with Aspirin for Evidence‐Based Pre‐eclampsia Prevention) trial and the FORECAST (Implementation of First‐Trimester Screening and Prevention of Pre‐eclampsia: A Stepped Wedge Cluster‐Randomized Trial in Asia) study reported a 62% (95% CI, 26–80%) and 41% (95% CI, 37–92%) reduction, respectively, in the incidence of preterm PE among high‐risk women receiving daily low‐dose aspirin, compared with those who received placebo or who did not receive aspirin, following first‐trimester screening using the Fetal Medicine Foundation (FMF) combined test^6^, ^7^.

Several theories have been proposed regarding the preventive effects of aspirin on PE, including improvement in trophoblastic invasion, endothelial stabilization and reduction in inflammation^8^, ^9^, ^10^. However, the effect of low‐dose aspirin on the maternal cardiovascular system remains unclear, and few studies have reported this^4^, ^11^. A study involving 35 women, 17 of whom received aspirin, found that antepartum aspirin therapy improved the global longitudinal strain in the second trimester, as assessed by transthoracic echocardiography (TTE)^11^. To date, only one longitudinal study has specifically investigated the effects of low‐dose aspirin on maternal hemodynamics in pregnancies that are high risk for preterm PE in the context of PE prevention, which reported that aspirin prophylaxis may not alter maternal hemodynamic profiles^4^. However, this study assessed maternal hemodynamics using a Non‐Invasive Cardiac Output Monitor (NICOM) in a predominantly European cohort, with only 2% of participants of Asian descent. Moreover, compared with two‐dimensional TTE (2D‐TTE), the reference standard method for monitoring hemodynamic circulation^12^, ^13^, the discrepancy in hemodynamic assessment between NICOM and 2D‐TTE remains inconsistent. Some studies reported acceptable agreement with discrepancies of less than 30%^14^, ^15^, whereas some showed discrepancies exceeding 30% across all trimesters^16^, ^17^. Given these inconsistencies, the applicability of the previous findings to East Asian populations remains uncertain. Differences in study populations and methodologies in earlier studies highlight the need for further investigation to ensure clinical relevance.

The objectives of this study were to longitudinally compare the maternal hemodynamics, measured using 2D‐TTE, between Chinese women at low risk and those at high risk for preterm PE, as well as between high‐risk Chinese women who received aspirin and those that did not.

## METHODS

### Study design and population

This prospective longitudinal case–control study was a substudy of the FORECAST study^7^, involving Chinese women with a singleton pregnancy who participated in the first‐trimester screen‐and‐prevent program for PE at the Prince of Wales Hospital, Hong Kong SAR, China, between February 2020 and March 2023. The study comprised two steps. First, routine PE screening was conducted for a population of women with a singleton pregnancy at 11 + 0 to 13 + 6 weeks' gestation using the FMF combined test, which combines maternal factors, MAP, uterine artery pulsatility index (UtA‐PI) and placental growth factor (PlGF). A cut‐off value of 1:100 was used to classify women as high risk (adjusted risk ≥ 1:100) or low risk (adjusted risk < 1:100) for preterm PE (delivery before 37 weeks' gestation). Second, the high‐risk women were invited at random to participate in the hemodynamic study and the low‐risk women were matched 1:1 with participating high‐risk women according to maternal age (within ± 3 years), weight (within ± 5 kg) and date of the scan (within ± 14 days).

The overall study cohort comprised three groups: Group 1, high‐risk women receiving a daily dose of 160 mg aspirin (100 mg if maternal weight < 40 kg) starting before 16 weeks' gestation until 36 weeks' gestation, or until delivery or the onset of PE before 36 weeks' gestation; Group 2, high‐risk women who declined aspirin treatment; and Group 3, matched low‐risk women. The dosage and duration of aspirin administration followed the recommendations of the International Society for the Study of Hypertension in Pregnancy (ISSHP)^18^ and the International Federation of Gynecology and Obstetrics (FIGO)^19^. Written informed consent was obtained from all participants and ethical approval was granted by the Joint Chinese University of Hong Kong–New Territories East Cluster Clinical Research Ethics Committee (reference number: 2016.292).

### Inclusion and exclusion criteria

The inclusion criteria were maternal age ≥ 18 years, singleton pregnancy with a viable fetus at 11 + 0 to 13 + 6 weeks of gestation, Chinese ethnicity, and high‐risk for preterm PE with or without prophylactic aspirin or matched low‐risk women. The exclusion criteria were multiple pregnancy, major fetal abnormality identified during the first‐trimester ultrasound, pre‐existing maternalcardiac disease except chronic hypertension (CH), inability to provide written informed consent or learning difficulties, termination of pregnancy or miscarriage before 24 weeks' gestation, and fewer than two clinical visits during the study period.

### Maternal factors and pregnancy outcomes

Maternal factors, including age, weight, height, racial origin (Chinese or other), smoking at the time of conception, mode of conception, parity (parous or nulliparous, defined as no previous pregnancy that reached or exceeded 24 weeks' gestation), history of PE in a previous pregnancy (parous with prior PE or parous without prior PE), family history of PE, CH, pre‐existing diabetes mellitus (DM), and systemic lupus erythematosus (SLE) or antiphospholipid syndrome (APS), were recorded at the time of preterm PE screening. Gestational age (GA), maternal weight and aspirin compliance were recorded during the subsequent clinical visits. Pregnancy outcomes were collected from the hospital maternity electronic records, including: GA at delivery; pregnancy complications, such as any‐onset PE (delivery at any GA), early‐onset PE (delivery before 34 weeks), preterm PE (delivery before 37 weeks), term PE (delivery at or after 37 weeks) and gestational hypertension (GH); mode of delivery (vaginal, instrumental or Cesarean section); neonatal birth weight; neonatal sex (male or female); and 1‐min Apgar score. PE and GH were defined according to the ISSHP guidelines^18^.

### Maternal hemodynamic measurement

Maternal hemodynamic parameters were measured and recorded at 12 + 0 to 15 + 6 weeks, 20 + 0 to 24 + 6 weeks and 30 + 0 to 37 + 6 weeks. Blood pressure (BP) was measured simultaneously in both arms at 1‐min intervals to obtain a total of four recordings using a validated automated device (BP 3AQ1; Microlife, Taipei, Taiwan). MAP was calculated using the following formula: MAP (mmHg) = diastolic BP + (systolic BP − diastolic BP) / 3^20^. Heart rate (HR) (in bpm), SV (in mL), CO (in L/min) and SVR (in dynes × s/cm^5^) were assessed using 2D‐TTE. Participants were required to reduce or avoid caffeine, tea and alcohol‐containing beverages for 24 h before the cardiac scan. To perform 2D‐TTE, a Voluson E6 (GE Healthcare, Zipf, Austria) ultrasound machine equipped with a wide‐band phased array transducer (3SP‐D; GE Healthcare) was used, according to the guidelines established by the American Society of Echocardiography^21^. 2D‐TTE was performed by two operators (X.W. and Y.C.) who were trained in the acquisition and analysis of echocardiograms by a cardiologist (A.P.W.L.) with expertise in echocardiography.

Our previous study reported good inter‐ and intraobserver reproducibility and repeatability of hemodynamic measurements^22^, and thus the same protocol was followed. The transducer was positioned in the fourth intercostal space, just to the left of the sternum, to visualize the parasternal long‐axis view of the heart. The left ventricular outflow tract diameter (LVOT‐D) was measured3–10 mm away from the aortic annulus using the inner‐edge‐to‐inner‐edge method during midsystole. Two replicated measurements were obtained, and the mean LVOT‐D was recorded. Subsequently, the cross‐sectional area of the LVOT‐D was calculated. After measuring the LVOT‐D, the transducer was positioned at the cardiac apex and tilted toward the chest to visualize the five‐chamber view of the heart. Pulsed‐wave Doppler was used, with the selected sample volume placed about 5 mm proximal to the aortic valves. The angle of insonation was maintained at less than 20°. When three similar consecutive waveforms were obtained, the left ventricular outflow tract velocity time integral (LVOT‐VTI) was measured for each waveform and the mean LVOT‐VTI was recorded. SV, CO and SVR were calculated based on the aforementioned measurements. SV was calculated using the formula SV = π × (LVOT‐D/2)^2^ × LVOT‐VTI; CO was calculated using the formula CO = SV × HR and SVR was calculated using the formula SVR = MAP/CO × 80. The results of maternal hemodynamic parameters were not disclosed to the participants or their doctors and did not influence the subsequent management of the pregnancy.

### Sample size

The sample size was calculated using the Glimmpse version 3.0.0 program (http://glimmpse.samplesizeshop.org/) for longitudinal studies based on the changes in CO observed during pregnancy among the three study groups (high‐risk for preterm PE with aspirin, high‐risk with placebo, low‐risk) reported in the study of Ling *et al*.^4^. At a power of 80% and a Type I error rate of 0.05, sample size estimation using the Lawley–Hotelling trace test indicated that a minimum of 84 women in each group was needed for our study. Considering a 20% loss‐to‐follow‐up rate and an 82% take‐up rate for aspirin, approximately 4167 women would need to be screened, with 14% expected to be identified as high‐risk^7^, ^23^.

### Statistical analysis

Maternal demographic characteristics, medical history and pregnancy outcomes were recorded for low‐risk and high‐risk women, with and without prophylactic aspirin. Categorical variables were compared using the chi‐square test or Fisher's exact test. Normality of the distribution of continuous data was assessed using the Kolmogorov–Smirnov test. As the data were not normally distributed, the distributions of HR, SV, CO, MAP and SVR were log_10_‐transformed to fit the data to a Gaussian distribution. For comparison of continuous data, the Kruskal–Wallis or one‐way ANOVA test with *post‐hoc* analysis were used for non‐normally and normally distributed data, respectively. Data were expressed as mean ± SD or median (interquartile range) for continuous variables. Categorical variables were expressed as *n* (%).

We performed multilevel linear mixed‐effects modeling for the repeated measures analysis of the maternal hemodynamic parameters, controlling for GA at each clinical visit (12 + 0 to 15 + 6 weeks, 20 + 0 to 24 + 6 weeks, 30 + 0 to 37 + 6 weeks), study group (high‐risk with aspirin, high‐risk without aspirin, low‐risk), maternal age, maternal weight, maternal height, history of PE in previous pregnancy, family history of PE, CH, pre‐existing DM, SLE or APS, mode of conception, smoking status, and the interaction between study group and clinical visits. The random effect components included the intercept (participant identity) and slope (clinical visit). The likelihood ratio test was used to define the optimal multilevel mixed‐effects model (fixed effect only, fixed effect + random slope, fixed effect + random intercept, and fixed effect + random intercept and slope), assuming an unstructured covariance structure for the random effects. Higher log‐likelihood values indicated a better model fit. The estimated marginal mean (EMmean) of each hemodynamic parameter at the three clinical visits was presented. To account for multiple comparisons, Bonferroni adjustment was applied to the *P*‐value across the pairwise tests; *P* < 0.05 was considered statistically significant.

The software program IBM SPSS version 28.0.1.0 (IBM Corp., Armonk, NY, USA) and R software (version 4.4.2, R Core Team, 2024; www.rstudio.org) were used for the statistical analysis. The dplyr package (version 1.1.4) was used for data processing and ggplot2 (version 3.5.1) was employed for data visualization. Mixed‐effects models were fitted using the lmer function from the lme4 package (version 1.1.35.5).

## RESULTS

### Study population

**Figure 1:**
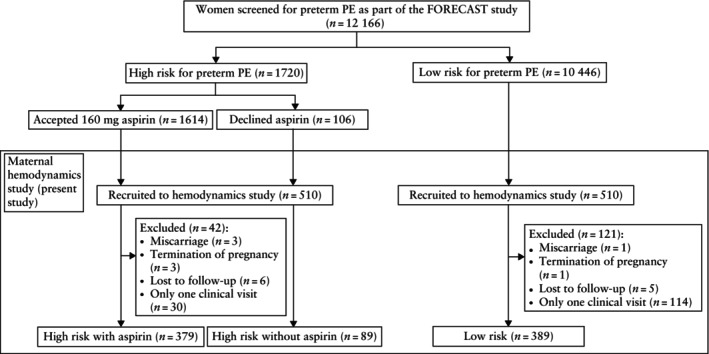
Flowchart showing inclusion in the study population of Chinese women with a singleton pregnancy from the FORECAST study^7^, who presented at the Prince of Wales Hospital, Hong Kong SAR, China. PE, pre‐eclampsia.

During the study period, a total of 12 166 women were screened in the first trimester for preterm PE risk, with 1720 (14.14%) women identified as being at high risk (Figure 1). Among these, 1614 (93.84%) women who accepted aspirin prophylaxis and 106 (6.16%) women who declined aspirin were approached to participate in the study. A total of 510 high‐risk women and 510 matched low‐risk women agreed to participate in this longitudinal study. We excluded 163 cases because of miscarriage (*n* = 4), termination of pregnancy for chromosomal or structural abnormalities (*n* = 4), loss to follow‐up (*n* = 11) and instances of only one clinical visit (*n* = 144). The final analysis included a cohort of 379 women at high risk for preterm PE who received aspirin, 89 high‐risk women who did not receive aspirin and 389 low‐risk women. Maternal clinical characteristics and pregnancy outcomes of the study population are presented in Table 1.

**Table 1:** Maternal clinical characteristics and pregnancy outcomes of women at low risk or at high risk for preterm pre‐eclampsia (PE) (delivery before 37 weeks' gestation) with or without prophylactic aspirin

	High risk					
Characteristic	Total (*n* = 468)	No aspirin (*n* = 89)	Aspirin (*n* = 379)	*P* †	Low risk (*n* = 389)	*P* ‡
Maternal age (years)	33.73 (31.17–37.11)	34.44 (31.50–37.01)	33.53 (31.02–37.12)	0.266	33.54 (30.99–36.04)	0.138
Maternal weight (kg)	58.90 (52.6–67.30)	58.80 (52.33–67.45)	59.00 (53.70–66.43)	0.825	56.00 (51.90–62.50)	0.003
Maternal height (cm)	159 (155–162)	159 (156–163)	159 (155–162)	0.432	160 (156–163)	< 0.001
Maternal BMI (kg/m^2^)	23.41 (21.12–26.48)	23.48 (21.78–26.49)	23.34 (20.99–26.48)	0.537	22.91 (20.13–23.92)	< 0.001
*In‐vitro* fertilization	45 (9.6)	13 (14.6)	32 (8.4)	0.076	32 (8.2)	0.479
Chronic hypertension	25 (5.3)	6 (6.7)	19 (5.0)	0.514	1 (0.3)	< 0.001
Pre‐existing diabetes mellitus	14 (3.0)	2 (2.2)	12 (3.2)	1.000	1 (0.3)	0.003
SLE/APS	7 (1.5)	1 (1.1)	6 (1.6)	1.000	0 (0)	0.018
Cigarette smoker	35 (7.5)	0	35 (9.2)	< 0.001	16 (4.1)	0.038
Family history of PE	8 (1.7)	1 (1.1)	7 (1.8)	1.000	1 (0.3)	0.045
Obstetric history				0.011		< 0.001
Nulliparous	334 (71.4)	68 (76.4)	266 (70.2)		227 (58.4)	
Parous, no prior PE	99 (21.2)	10 (11.2)	89 (23.5)		157 (40.4)	
Parous, prior PE	35 (7.5)	11 (12.4)	24 (6.3)		5 (1.3)	
Adjusted risk for preterm PE (1:xx)	48 (23–73)	45 (19–73)	48 (24–73)	0.630	710 (276–1788)	< 0.001
Aspirin compliance (%)	99.35 (96.79–100.00)	—	99.35 (96.79–100.00)	—	—	—
MAP MoM at screening*	1.10 (1.03–1.18)	1.07 (0.98–1.13)	1.11 (1.05–1.18)	< 0.001	1.00 (0.95–1.06)	< 0.001
UtA‐PI MoM at screening*	1.20 (1.03–1.39)	1.22 (1.02–1.41)	1.20 (1.03–1.39)	0.602	1.00 (0.83–1.16)	< 0.001
PlGF MoM at screening*	0.59 (0.42–0.79)	0.55 (0.38–0.71)	0.61 (0.42–0.82)	0.049	1.04 (0.81–1.33)	< 0.001
Pregnancy outcome						
GA at delivery (days)	268 (263–274)	267 (261–276)	268 (263–274)	0.684	272 (267–279)	< 0.001
Birth weight (g)	2930 (2630–3160)	2965 (2617–3172)	2925 (2640–3160)	0.999	3113 (2855–3415)	<0.001
Mode of delivery				0.924		0.006
Vaginal	218 (46.6)	42 (47.2)	176 (46.4)		222 (57.1)	
Instrumental	28 (6.0)	6 (6.7)	22 (5.8)		24 (6.2)	
Cesarean section	222 (47.4)	41 (46.1)	181 (47.8)		143 (36.8)	
Fetal sex				0.634		0.403
Male	242 (51.7)	44 (49.4)	198 (52.2)		190 (48.8)	
Female	226 (48.3)	45 (50.6)	181 (47.8)		199 (51.2)	
1‐min Apgar score < 7	32 (6.8)	6 (6.7)	26 (6.9)	0.818	7 (1.8)	< 0.001
Pregnancy complications						
Gestational hypertension	27 (5.8)	5 (5.6)	22 (5.8)	0.946	4 (1.0)	< 0.001
PE	45 (9.6)	8 (9.0)	37 (9.8)	0.842	3 (0.8)	< 0.001
Delivery < 34 weeks	11 (2.4)	2 (2.2)	9 (2.4)	1.000	2 (0.5)	0.045
Delivery < 37 weeks	31 (6.6)	6 (6.7)	25 (6.6)	0.960	2 (0.5)	< 0.001
Delivery ≥ 37 weeks	14 (3.0)	2 (2.2)	12 (3.2)	1.000	1 (0.3)	0.003

Data are given as median (interquartile range) or *n* (%).

* Gestational age at screening was between 11 + 0 and 13 + 6 weeks.

† High risk with aspirin *vs* high risk without aspirin.

‡ Low risk *vs* high risk. APS, antiphospholipid syndrome; BMI, body mass index; GA, gestational age; MAP, mean arterial pressure; MoM, multiples of the median; PlGF, placental growth factor; SLE, systemic lupus erythematosus; UtA‐PI, uterine artery pulsatility index.

In comparison to low‐risk women, high‐risk women had higher BMI, lower height and exhibited higher MAP multiples of the median (MoM) and UtA‐PI MoM values, as well as lower PlGF MoM values (*P* < 0.01 for all). The high‐risk group also had higher proportions of cigarette smokers, nulliparous individuals, women who were parous with a history of prior PE, CH, pre‐existing DM, SLE/APS, and a family history of PE (all *P* < 0.05). Regarding pregnancy outcomes, high‐risk women delivered at an earlier GA, had neonates with lower birth weight, and had higher rates of 1‐min Apgar score < 7, Cesarean section and pregnancy complications including PE and GH (all *P* < 0.01). When comparing high‐risk women who received aspirin to those who did not, the group that received aspirin had a significantly greater proportion of cigarette smokers and women with family history or PE, as well as significantly higher MAP MoM and lower PlGF MoM values (all *P* < 0.05); however, there were no significant differences in neonatal birth weight, GA at delivery, Cesarean section rate, 1‐min Apgar score < 7 and pregnancy complications.

### Temporal changes between study groups across gestation

**Figure 2:**
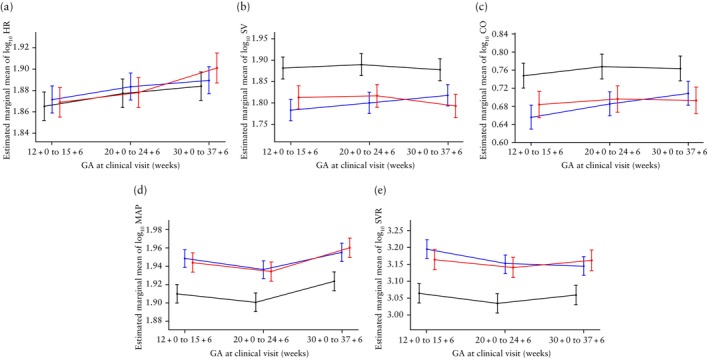
Temporal changes in estimated marginal mean ± standard error of log_10_ heart rate (HR) (a), log_10_ stroke volume (SV) (b), log_10_ cardiac output (CO) (c), log_10_ mean arterial pressure (MAP) (d) and log_10_ systemic vascular resistance (SVR) (e) across gestation. 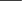, low risk for preterm pre‐eclampsia (PE); 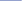, high risk for preterm PE with aspirin; 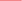, high‐risk for preterm PE without aspirin. GA, gestational age.

The results of the multilevel mixed‐effects model for log_10_ HR across gestation, along with the best‐fit model, are shown in Table S1 and Appendix S1, respectively.  The EMmean of log_10_ HR values demonstrated a significant upward trend with advancing gestation, for all study groups (all *P* < 0.001). However, there were no significant differences in the EMmean of log_10_ HR between the three study groups throughout gestation (Table 2, Figure 2a).

**Table 2:** Comparisons of estimated marginal mean (EMmean) of log_10_ hemodynamic parameters within and between groups of pregnant women at low risk or high risk for preterm pre‐eclampsia (delivery before 37 weeks' gestation) across gestation

	12 + 0 to 15 + 6 weeks				20 + 0 to 24 + 6 weeks				30 + 0 to 37 + 6 weeks				
Study groups	*n*	EMmean	SE	95% CI	*n*	EMmean	SE	95% CI	*n*	EMmean	SE	95% CI	*P* (within groups)
Log_10_ HR													
Low‐risk	389	1.86521	0.01326	1.83919–1.89123	389	1.87719	0.01325	1.85119–1.90318	366	1.88396	0.01327	1.85792–1.91001	< 0.001‡, §
High‐risk with aspirin	379	1.87139	0.01272	1.84642–1.89636	376	1.88362	0.01271	1.85867–1.90857	327	1.88946	0.01278	1.86438–1.91454	< 0.001‡, §
High‐risk without aspirin	89	1.86866	0.01384	1.84150–1.89583	88	1.87805	0.01380	1.85097–1.90512	78	1.90097	0.01401	1.87347–1.92846	< 0.001§, ¶
*P* (between groups)	> 0.05				> 0.05				> 0.05				
Log_10_ SV													
Low‐risk	389	1.88171	0.02581	1.83105–1.93237	389	1.88968	0.02579	1.83905–1.94030	366	1.87798	0.02587	1.82720–1.92876	> 0.05
High‐risk with aspirin	379	1.78356	0.02477	1.73495–1.83218	376	1.80034	0.02476	1.75175–1.84893	327	1.81777	0.02493	1.76884–1.86671	< 0.05‡, §, ¶
High‐risk without aspirin	89	1.81330	0.02697	1.76037–1.86622	88	1.81665	0.02692	1.76382–1.86947	78	1.79303	0.02752	1.73903–1.84703	> 0.05
*P* (between groups)	< 0.001*, †				< 0.001*, †				< 0.001*, †				
Log_10_ CO													
Low‐risk	389	0.74758	0.02761	0.69339–0.80178	389	0.76792	0.02759	0.71377–0.82206	366	0.76333	0.02766	0.70905–0.81761	< 0.01‡
High‐risk with aspirin	379	0.65583	0.02650	0.60382–0.70784	376	0.68564	0.02648	0.63367–0.73761	327	0.70847	0.02666	0.65615–0.76080	< 0.001‡, §, ¶
High‐risk without aspirin	89	0.68377	0.02894	0.62698–0.74057	88	0.69600	0.02885	0.63938–0.75262	78	0.69299	0.02947	0.63517–0.75080	> 0.05
*P* (between groups)	< 0.001*, †				< 0.001*, †				< 0.001*, †				
Log_10_ MAP													
Low‐risk	389	1.90932	0.01026	1.88918–1.92945	389	1.90009	0.01025	1.87998–1.92020	366	1.92308	0.01027	1.90291–1.94324	< 0.001‡, §, ¶
High‐risk with aspirin	379	1.94810	0.00984	1.92878–1.96741	376	1.93591	0.00983	1.91661–1.95520	327	1.95481	0.00988	1.93542–1.97420	< 0.01‡, §, ¶
High‐risk without aspirin	89	1.94377	0.01061	1.92295–1.96459	88	1.93369	0.01056	1.91296–1.95442	78	1.95985	0.01073	1.93880–1.98091	< 0.01‡, §, ¶
*P* (between groups)	< 0.001*, †				< 0.001*, †				< 0.001*, †				
Log_10_ SVR													
Low‐risk	389	3.06408	0.02863	3.00789–3.12027	389	3.03431	0.02861	2.97816–3.09045	366	3.05902	0.02870	3.00268–3.11535	< 0.001‡, ¶
High‐risk with aspirin	379	3.19506	0.02748	3.14114–3.24889	376	3.15264	0.02746	3.09875–3.20653	327	3.14495	0.02767	3.09065–3.19924	< 0.001‡, §
High‐risk without aspirin	89	3.16390	0.02994	3.10514–3.22266	88	3.14027	0.02987	3.08211–3.19933	78	3.16185	0.03058	3.10185–3.22186	> 0.05
*P* (between groups)	< 0.001*, †				< 0.001*, †				< 0.001*, †				

* High risk with aspirin *vs* Low risk;

† High risk without aspirin *vs* Low risk;

‡ 12 + 0 to 15 + 6 weeks *vs* 20 + 0 to 24 + 6 weeks;

§ 12 + 0 to 15 + 6 weeks *vs* 30 + 0 to 37 + 6 weeks;

¶ 20 + 0 to 24 + 6 weeks *vs* 30 + 0 to 37 + 6 weeks. Significance value adjusted by the Bonferroni correction for multiple tests. CO, cardiac output; HR, heart rate; MAP, mean arterial pressure; SE, standard error; SV, stroke volume; SVR, systemic vascular resistance.

The results of the multilevel mixed‐effects model for the log_10_ SV across gestation, along with the best‐fit model, are shown in Table S2 and Appendix S1, respectively. The low‐risk group maintained the highest EMmean of log_10_ SV, with only minor fluctuations throughout gestation. The high‐risk with aspirin group showed a gradual increase in EMmean of log_10_ SV across gestation (*P* < 0.05), while the high‐risk without aspirin group showed a slight increase in EMmean of log_10_ SV at 20 + 0 to 24 + 6 weeks, followed by a subsequent decrease (Figure 2b, Table 2). Across gestation, the high‐risk groups consistently demonstrated a significantly lower EMmean of log_10_ SV compared with the low‐risk group (all *P* < 0.001), but no significant difference in EMmean of log_10_ SV was observed between the two high‐risk groups.

The results of the multilevel mixed‐effects model for the log_10_ CO across gestation, along with the best‐fit model, are shown in Table S3 and Appendix S1, respectively. The low‐risk group maintained the highest EMmean of log_10_ CO across gestation, and there was a significant change between 12 + 0 to 15 + 6 weeks and 20 + 0 to 24 + 6 weeks (*P* < 0.01). The high‐risk with aspirin group showed a significant and progressive increase in EMmean of log_10_ CO across gestation, peaking at 30 + 0 to 37 + 6 weeks (all *P* < 0.001), while the high‐risk without aspirin group showed a slight increase in EMmean of log_10_ CO at 20 + 0 to 24 + 6 weeks, plateauing in late gestation (Figure 2c, Table 2). Across gestation, the high‐risk groups consistently exhibited a lower EMmean of log_10_ CO compared with the low‐risk group (all *P* < 0.001), but no significant difference in EMmean of log_10_ CO was observed between the two high‐risk groups.

The results of the multilevel mixed‐effects model for the log_10_ MAP across gestation, along with the best‐fit model, are shown in Table S4 and Appendix S1, respectively. The EMmean of log_10_ MAP exhibited a V‐shaped trajectory, declining from early to mid‐gestation, followed by an increase in late gestation for all study groups (all *P* < 0.01) (Figure 2d, Table 2). Across gestation, the high‐risk groups consistently exhibited higher EMmean of log_10_ MAP compared with the low‐risk group (all *P* < 0.001). The high‐risk with aspirin group exhibited a slightly higher EMmean of log_10_ MAP at 12 + 0 to 15 + 6 and 20 + 0 to 24 + 6 weeks, but a lower value at 30 + 0 to 37 + 6 weeks compared with the high‐risk without aspirin group. Nevertheless, no significant difference in the EMmean of log_10_ MAP was observed between the two high‐risk groups.

The results of the multilevel mixed‐effects model for the log_10_ SVR across gestation, along with the best‐fit model, are shown in Table S5 and Appendix S1, respectively. The low‐risk group and high‐risk without aspirin group exhibited a U‐shaped trajectory in EMmean of log_10_ SVR, decreasing during mid‐gestation and returning to early pregnancy levels in late gestation. In contrast, the high‐risk with aspirin group showed a significant gradual reduction in EMmean of log_10_ SVR from early to late gestation (*P* < 0.001) (Figure 2e, Table 2). Across gestation, the high‐risk groups consistently exhibited higher EMmean of log_10_ SVR compared with the low‐risk group (all *P* < 0.001), but no significant difference in EMmean of log_10_ SVR was observed between the two high‐risk groups.

## DISCUSSION

### Principal findings

This study, which used 2D‐TTE as the reference standard for monitoring hemodynamic circulation, has provided key insights into maternal hemodynamic maladaptation in pregnancies at risk for preterm PE (delivery before 37 weeks' gestation), along with the limited role of aspirin prophylaxis in modifying these changes. First, high‐risk women exhibited significantly impaired cardiovascular adaptations to the physiological demands of pregnancy as early as the first trimester, characterized by persistently increased MAP and SVR, along with decreased CO and SV throughout gestation compared with low‐risk women. Second, maternal hemodynamic trajectories of SV, CO, MAP and SVR differed between high‐risk women who received aspirin and those who did not; however, no statistically significant differences in the EMmean values were observed throughout gestation between the two high‐risk groups. This may suggest that aspirin prophylaxis does not significantly normalize maternal hemodynamic parameters across gestation.

### Results in the context of existing literature

Our findings align with previous research indicating that women at high risk for preterm PE are predisposed to impaired cardiovascular adaptation^4^, ^24^, ^25^, ^26^, ^27^, ^28^, ^29^, ^30^ and adverse pregnancy outcomes^6^, ^24^, ^31^. In our study, high‐risk women consistently exhibited significantly higher MAP and SVR and lower SV and CO compared with low‐risk women, irrespective of aspirin prophylaxis during pregnancy. These results are consistent with previous studies that reported high‐risk women exhibit significant hemodynamic dysregulation during pregnancy^4^, ^29^, ^30^. In addition, high‐risk women experienced poorer pregnancy outcomes, including delivery at an earlier GA, neonates with lower birth weight and lower Apgar scores, as well as higher rates of Cesarean section and pregnancy complications such as PE and GH. Despite its known efficacy in reducing the incidence of preterm PE^6^, ^7^, aspirin did not significantly normalize hemodynamic parameters in high‐risk women in our study. Although the trajectories of SV, CO, MAP and SVR differed between high‐risk women who received aspirin and those who did not, these differences did not translate into statistically significant improvements in hemodynamic parameters. This suggests that aspirin has minimal impact on maternal cardiovascular function and its protective effects are exerted through other mechanisms, such as improved placental perfusion^29^. Beyond its limited hemodynamic effects, low‐dose aspirin is also known to exert anti‐inflammatory^32^, ^33^, anti‐platelet^32^, ^34^ and anti‐angiogenic^35^ actions, and these pleiotropic effects may also play a role in reducing the risk of PE^36^. Future research should explore these alternative pathways to better understand the mechanism of action of aspirin in pregnancy.

Interestingly, the longitudinal changes in SV and CO observed in our study differed from those reported in the study of Ling *et al*.^4^, in which the study population primarily consisted of White women (79%), whereas our cohort was composed of Chinese women only. Previously, the same research group reported race‐specific differences in maternal cardiac adaptation during pregnancy, noting that SV increased initially in early pregnancy but subsequently declined with GA in White women, while remaining static in Asian women^31^. Additionally, the proportion of high‐risk women with specific risk factors, such as CH, SLE/APS and DM, which are known risk factors for worsening the maternal cardiac function^19^, ^37^, ^38^, was higher in the study of Ling *et al*.^4^ compared with ours. Furthermore, Ling *et al*.^4^ assessed maternal hemodynamics using NICOM, whereas our study used 2D‐TTE. Our previous study^14^ demonstrated that NICOM tends to underestimate maternal hemodynamic parameters compared with 2D‐TTE, with the mean percentage difference exceeding 70% in mid‐ and late gestation among Chinese women. These methodological differences may contribute to the discrepancies in hemodynamic trajectories observed between our study and that of Ling *et al*.^4^

### Clinical and research implications

This study highlights the critical importance of understanding maternal hemodynamic maladaptation in high‐risk pregnancies. First, hemodynamic assessment could play an important role in identifying cardiovascular maladaptation in women at high risk for preterm PE. Early identification of hemodynamic maladaptation may guide the risk stratification and targeted clinical decision‐making. Integrating non‐invasive hemodynamic monitoring, such as TTE, NICOM or ultrasonographic cardiac output monitors, into prenatal care could enhance the early detection of deteriorating cardiovascular function, potentially improving both maternal and fetal outcomes. Although TTE is considered a reference standard for cardiac imaging because of its high spatial resolution and diagnostic reliability^39^, its routine application during pregnancy is limited by its invasiveness and accessibility. Second, although aspirin continues to be fundamental in PE prevention, its limited impact on maternal hemodynamics highlights the need for additional strategies to optimize cardiovascular function. Exploring complementary interventions that enhance vascular adaptation may further improve both maternal and neonatal outcomes. Moreover, the mechanisms underlying the effects of aspirin on maternal hemodynamics, particularly in relation to placental function and vascular remodeling, warrant further investigation. A deeper understanding of these pathways could inform the development of novel therapeutic targets to mitigate hemodynamic maladaptation in high‐risk pregnancies.

### Strengths and limitations

Our study has several strengths. First, to our knowledge, this is the first study with a relatively large sample size to investigate the effects of aspirin on maternal hemodynamics using reference‐standard 2D‐TTE, incorporating a detailed temporal analysis of maternal hemodynamic trajectories throughout pregnancy. Second, the use of multilevel mixed‐effects models enhanced the analysis by enabling precise estimation of hemodynamic changes over time while accounting for within‐subject variability. Furthermore, by including women who attended a minimum of two clinical visits and ensuring the missing data rate remained below 10%, we further strengthened the robustness of our findings. Moreover, we employed a well‐validated and highly accurate screening method to identify women at risk of developing preterm PE, which has been endorsed by leading international professional organizations for clinical practice^19^, ^23^, ^40^.

However, certain limitations of our study should be acknowledged. First, the administration of prophylactic aspirin has been a standard clinical practice for women at risk for preterm PE in our unit since 2020, which has resulted in only a relatively small number of high‐risk women not receiving aspirin in our study. Nonetheless, we successfully achieved the target sample size for each study group, ensuring sufficient statistical power. Second, maternal weight was matched between the study groups at recruitment, but significant differences still existed, which were addressed using the linear mixed‐effects model during the analysis to account for potential confounders. Third, because of the limited statistical power, we did not assess associations between maternal hemodynamics and pregnancy outcomes; however, we acknowledge that exploring these associations is a valuable direction for future research.

### Conclusions

This study highlights the significant differences in maternal hemodynamic adaptations during pregnancy between high‐risk and low‐risk women for preterm PE within a Chinese population. Compared with low‐risk women, high‐risk women exhibited increased MAP and SVR, along with reduced SV and CO as early as the first trimester, and these alterations persisted throughout gestation. Notably, while aspirin prophylaxis was associated with altered hemodynamic trajectories across gestation, it had a limited effect on improving maternal hemodynamics in women at high risk for preterm PE, highlighting the need for alternative strategies to address the hemodynamic maladaptation in high‐risk women.

## Supporting information


**Appendix S1** Multilevel mixed‐effects model selection.
**Tables S1–S5** Multilevel mixed‐effects models of the median log_10_ heart rate (Table S1), stroke volume (Table S2), cardiac output (Table S3), mean arterial pressure (Table S4) and systemic vascular resistance (Table S5) across gestation, showing fixed effects.

## Data Availability

The data that support the findings of this study are available from the corresponding author upon reasonable request.
