# Supplementary material for: Effect of aspirin on maternal hemodynamics in Chinese women at high risk for preterm pre‐eclampsia: longitudinal study
Source: Ultrasound Obstet Gynecol. 2025 Sep 12;66(5):621–30. doi: 10.1002/uog.70027 (PMC12579775; doi:10.1002/uog.70027)
Supplement: Supplementary file 1 — Appendix S1 Multilevel mixed‐effects model selection. Tables S1–S5 Multilevel mixed‐effects models of the median log10 heart rate (Table S1), stroke volume (Table S2), cardiac output (Table S3), mean arterial pressure (Table S4) and systemic vascular resistance (Table S5) across gestation, showing fixed effects. [file UOG-66-621-s001.docx]

Supplementary

Appendix S1. Mixed-effects model selection

**Mixed-effects model selection of log10 heart rate**

A mixed-effects model analysis showed that the log-likelihood values for Model 1 (fixed effects only), Model 2 (fixed effects + random intercept), Model 3 (fixed effects + random slope), and Model 4 (fixed effects + random intercept & slope) were 3742.8, 3944.8, 3867.2, and 3947.4, respectively. The higher the log-likelihood value, the better the model fit. Model 4 had the highest log-likelihood value and was identified to be the best-fitting model.

**Mixed-effects model selection of log_10_ stroke volume**

A mixed-effects model analysis showed that the log-likelihood values for Model 1 (fixed effects only), Model 2 (fixed effects + random intercept), Model 3 (fixed effects + random slope), and Model 4 (fixed effects + random intercept & slope) were 2043.4, 2209.4, 2153.8, and 2213.7, respectively. Model 4 had the highest log-likelihood value and was identified to be the best-fitting model.

**Mixed-effects model selection of log_10_ cardiac output**

A mixed-effects model analysis showed that the log-likelihood values for Model 1 (fixed effects only), Model 2 (fixed effects + random intercept), Model 3 (fixed effects + random slope), and Model 4 (fixed effects + random intercept & slope) were 1839.2, 1976.3, 1925.8, and 1979.2, respectively. Model 4 had the highest log-likelihood value and was identified to be the best-fitting model.

**Mixed-effects model selection of log_10_ mean arterial pressure**

A mixed-effects model analysis showed that the log-likelihood values for Model 1 (fixed effects only), Model 2 (fixed effects + random intercept), Model 3 (fixed effects + random slope), and Model 4 (fixed effects + random intercept & slope) were 4579.0, 4992.9, 4830.7, and 5010.9, respectively. Model 4 had the highest log-likelihood value and was identified to be the best-fitting model.

**Mixed-effects model selection of of log_10_ systemic vascular resistance**

A mixed-effects model analysis showed that the log-likelihood values for Model 1 (fixed effects only), Model 2 (fixed effects + random intercept), Model 3 (fixed effects + random slope), and Model 4 (fixed effects + random intercept & slope) were 1772.0, 1926.0, 1875.7, and 1931.4, respectively. Model 4 had the highest log-likelihood value and was identified to be the best-fitting model.

Table S1. Multilevel mixed effect model of the median log_10_ heart rate across gestation: fixed effects.

| Independent variables | Coefficient | Standard error | 95% confidence interval | p value |
| --- | --- | --- | --- | --- |
| Intercept | 2.01700 | 0.04655 | 1.92576 to 2.10824 | <0.001 |
| Maternal height (cm) | 0.01086 | 0.02930 | -0.04651 to 0.06823 | <0.001 |
| Maternal weight (kg) | 0.06907 | 0.01680 | 0.03589 to 0.10225 | <0.001 |
| Clinical visits (week) |  |  |  |  |
| Visit 1 (12^+0^–15^+6^ ) | - | - | - | - |
| Visit 2 (20^+0^–24^+6^) | 0.01198 | 0.02846 | -0.04392 to 0.06788 | <0.001 |
| Visit 3 (30^+0^–37^+6^) | 0.01876 | 0.03080 | -0.04142 to 0.07894 | <0.001 |

Only independent variables with p <0.05 were demonstrated.

Table S2. Multilevel mixed effect model of the median log_10_ stroke volume across gestation: fixed effects.

| Independent variables | Coefficient | Standard error | 95% confidence interval | p value |
| --- | --- | --- | --- | --- |
| Intercept | 1.47600 | 0.09067 | 1.29729 to 1.65471 | <0.001 |
| Maternal height (cm) | 0.00150 | 0.00057 | 0.00038 to 0.00262 | 0.009 |
| Maternal weight (kg) | 0.00210 | 0.00032 | 0.00147 to 0.00273 | <0.001 |
| Past obstetric history |  |  |  |  |
| Nulliparous | - | - | - | - |
| Parous, no prior PE | 0.02085 | 0.00681 | 0.00749 to 0.03421 | 0.002 |
| Parous, prior PE | 0.02522 | 0.01404 | -0.00229 to 0.05273 | 0.073 |
| Study groups |  |  |  |  |
| Low-risk | - | - | - | - |
| High-risk with aspirin | -0.09814 | 0.00805 | -0.11391 to -0.08237 | <0.001 |
| High-risk without aspirin | -0.06841 | 0.01291 | -0.09372 to -0.04310 | <0.001 |
| Clinical visits * study groups |  |  |  |  |
| Visit 2 (20^+0^–24^+6^) * high-risk with aspirin | 0.00881 | 0.00823 | -0.00734 to 0.02496 | 0.285 |
| Visit 3 (30^+0^–37^+6^) * high-risk with aspirin | 0.03794 | 0.00919 | 0.01993 to 0.05595 | <0.001 |
| Visit 2 (20^+0^–24^+6^) * high-risk without aspirin | -0.00462 | 0.01343 | -0.03095 to 0.02171 | 0.731 |
| Visit 3 (30^+0^–37^+6^) * high-risk without aspirin | -0.01654 | 0.01508 | -0.04610 to 0.01302 | 0.273 |

Only independent variables with p <0.05 were demonstrated.

Table S3. Multilevel mixed effect model of the median log_10_ cardiac output across gestation: fixed effects.

| Independent variables | Coefficient | Standard error | 95% confidence interval | p value |
| --- | --- | --- | --- | --- |
| Intercept | 0.49980 | 0.09671 | 0.30924 to 0.69036 | <0.001 |
| Maternal weight (kg) | 0.00278 | 0.00035 | 0.00209 to 0.00347 | <0.001 |
| Past obstetric history |  |  |  |  |
| Nulliparous | - | - | - | - |
| Parous, no prior PE | 0.02396 | 0.00727 | 0.00972 to 0.03820 | 0.001 |
| Parous, prior PE | 0.02801 | 0.01499 | -0.00130 to 0.05732 | 0.062 |
| Clinical visits (week) |  |  |  |  |
| Visit 1 (12^+0^–15^+6^ ) | - | - | - | - |
| Visit 2 (20^+0^–24^+6^) | 0.02033 | 0.00673 | 0.00714 to 0.03352 | 0.002 |
| Visit 3 (30^+0^–37^+6^) | 0.01575 | 0.00702 | 0.00197 to 0.02953 | 0.025 |
| Study groups |  |  |  |  |
| Low-risk | - | - | - | - |
| High-risk with aspirin | -0.09175 | 0.00880 | -0.10899 to -0.07451 | <0.001 |
| High-risk without aspirin | -0.06381 | 0.01411 | -0.09147 to -0.03615 | <0.001 |
| Clinical visits * study groups |  |  |  |  |
| Visit 2 (20^+0^–24^+6^) * high-risk with aspirin | 0.00948 | 0.00923 | -0.00860 to 0.02756 | 0.305 |
| Visit 3 (30^+0^–37^+6^) * high-risk with aspirin | 0.03690 | 0.01017 | 0.01696 to 0.05684 | <0.001 |
| Visit 2 (20^+0^–24^+6^) * high-risk without aspirin | -0.00811 | 0.01505 | -0.03761 to 0.02139 | 0.590 |
| Visit 3 (30^+0^–37^+6^) * high-risk without aspirin | -0.00653 | 0.01669 | -0.03930 to 0.02624 | 0.696 |

Only independent variables with p <0.05 were demonstrated.

Table S4. Multilevel mixed effect model of the median log_10_ mean arterial pressure across gestation: fixed effects.

| Independent variables | Coefficient | Standard error | 95% confidence interval | p value |
| --- | --- | --- | --- | --- |
| Intercept | 1.87600 | 0.03633 | 1.80478 to 1.94722 | <0.001 |
| Maternal height (cm) | -0.00055 | 0.00023 | -0.00100 to -0.00010 | 0.016 |
| Maternal weight (kg) | 0.00173 | 0.00013 | 0.00147 to 0.00199 | <0.001 |
| Cigarette smoker | -0.01293 | 0.00497 | -0.02268 to -0.00318 | 0.009 |
| Chronic hypertension | 0.04877 | 0.00723 | 0.03462 to 0.06292 | <0.001 |
| Past obstetric history |  |  |  |  |
| Nulliparous | - | - |  | - |
| Parous, no prior PE | -0.00123 | 0.00273 | -0.00659 to 0.00413 | 0.652 |
| Parous, prior PE | 0.01654 | 0.00562 | 0.00552 to 0.02756 | 0.003 |
| Clinical visits (week) |  |  |  |  |
| Visit 1 (12^+0^–15^+6^ ) | - | - |  | - |
| Visit 2 (20^+0^–24^+6^) | -0.00923 | 0.00165 | -0.01247 to -0.00600 | <0.001 |
| Visit 3 (30^+0^–37^+6^) | 0.01376 | 0.00195 | 0.00994 to 0.01758 | <0.001 |
| Study groups |  |  |  |  |
| Low-risk | - | - |  | - |
| High-risk with aspirin | 0.03878 | 0.00297 | 0.03294 to 0.04462 | <0.001 |
| High-risk without aspirin | 0.03445 | 0.00474 | 0.02516 to 0.04374 | <0.001 |
| Clinical visits * study groups |  |  |  |  |
| Visit 2 (20^+0^–24^+6^) * high-risk with aspirin | -0.00296 | 0.00235 | -0.00757 to 0.00165 | 0.207 |
| Visit 3 (30^+0^–37^+6^) * high-risk with aspirin | -0.00704 | 0.00282 | -0.01257 to -0.00151 | 0.013 |
| Visit 2 (20^+0^–24^+6^) * high-risk without aspirin | -0.00085 | 0.00382 | -0.00834 to 0.00664 | 0.823 |
| Visit 3 (30^+0^–37^+6^) * high-risk without aspirin | 0.00232 | 0.00461 | -0.00673 to 0.01137 | 0.614 |

Only independent variables with p <0.05 were demonstrated.

Table S5. Multilevel mixed effect model of the median log_10_ systemic vascular resistance across gestation: fixed effects.

| Independent variables | Coefficient | Standard error | 95% confidence interval | p value |
| --- | --- | --- | --- | --- |
| Intercept | 3.28400 | 0.00036 | 3.28329 to 3.28471 | <0.001 |
| Maternal weight (kg) | -0.00108 | 0.00035 | -0.00178 to -0.00038 | 0.003 |
| Past obstetric history |  |  |  |  |
| Nulliparous | - | - |  | - |
| Parous, no prior PE | -0.00247 | 0.00755 | -0.01728 to 0.01234 | 0.001 |
| Parous, prior PE | -0.01174 | 0.01577 | -0.04266 to 0.01918 | 0.451 |
| Clinical visits (week) |  |  |  |  |
| Visit 1 (12^+0^–15^+6^ ) | - | - |  | - |
| Visit 2 (20^+0^–24^+6^) | -0.02977 | 0.00650 | -0.04254 to -0.01700 | <0.001 |
| Visit 3 (30^+0^–37^+6^) | -0.00506 | 0.00719 | -0.01917 to 0.00905 | <0.001 |
| Study groups |  |  |  |  |
| Low-risk | - | - |  | - |
| High-risk with aspirin | 0.13100 | 0.00899 | 0.11337 to 0.14863 | <0.001 |
| High-risk without aspirin | 0.09982 | 0.01441 | 0.07155 to 0.12809 | <0.001 |
| Clinical visits * study groups |  |  |  |  |
| Visit 2 (20^+0^–24^+6^) * high-risk with aspirin | -0.01265 | 0.00926 | -0.03079 to 0.00549 | 0.172 |
| Visit 3 (30^+0^–37^+6^) * high-risk with aspirin | -0.04505 | 0.01041 | -0.06546 to -0.02464 | <0.001 |
| Visit 2 (20^+0^–24^+6^) * high-risk without aspirin | 0.00659 | 0.01510 | -0.02301 to 0.03619 | 0.663 |
| Visit 3 (30^+0^–37^+6^) * high-risk without aspirin | 0.00302 | 0.01709 | -0.03049 to 0.03653 | 0.860 |

Only independent variables with p <0.05 were demonstrated.
